# Analysis of Multi-Pesticide Residues and Dietary Risk Assessment in Fresh Tomatoes (*Lycopersicum esculentum*) from Local Supermarkets of the Metropolitan Region, Chile

**DOI:** 10.3390/toxics9100249

**Published:** 2021-10-06

**Authors:** Sebastian Elgueta, Marcela Valenzuela, Marcela Fuentes, Pilar E. Ulloa, Cecilia Ramos, Arturo Correa, Sebastian Molinett

**Affiliations:** 1 Núcleo de Investigaciones Aplicadas en Ciencias Veterinarias y Agronómicas, Universidad de Las Américas, Sede Providencia, Santiago 7500975, Chile; pilar.ulloa@udla.cl (P.E.U.); cramos@udla.cl (C.R.); 2 Laboratory of Pesticide Residues, Instituto de Investigaciones Agropecuarias, La Platina, Santiago 8720000, Chile; marcela.valenzuela@inia.cl (M.V.); mfuentes@inia.cl (M.F.); arturo.correabriones@gmail.com (A.C.); 3 Bionanotechnology Department, Instituto de Investigaciones Agropecuarias La Cruz, La Cruz 2280454, Chile; smolinett@gmail.com

**Keywords:** pesticide, risk assessment, tomato, methamidophos, chlorpyrifos, methomyl

## Abstract

In recent years, the official authorities in Chile have reported transgressions in the maximum residue levels of pesticides in fresh vegetables. There is no official information about traceability, pesticide levels, and potential health risks. The aim of this study was to analyse pesticide residues and their corresponding dietary risk assessments in tomatoes from supermarkets in the Metropolitan Region. Pesticides were extracted using the Quick, Easy, Cheap, Effective, Rugged and Safe, QuEChERS method, and their concentrations were determined by using chromatography with HPLC-FL/UV and GC-MS/ECD/NPD, following the Analytical Quality Control and Method Validation Procedures for Pesticides Residues Analysis in Food and Feed, SANTE guide and ISO 17025:2017 standard. In addition, a dietary risk assessment was carried out by comparing Chilean data to international references. The results reported that 9% of the samples had pesticide residue levels above the maximum residue levels permitted in Chile. All the scenarios evaluated revealed the highest estimated daily intake and hazard quotients for methamidophos and chlorpyrifos. Both the active substances used were acetylcholinesterase inhibitors and were neurotoxic under chronic risk assessment. The results showed the highest chronic hazard index in the Chilean scenario for all age groups and genders. The evidence obtained revealed that methamidophos, methomyl, and chlorpyrifos should be restricted for their use in Chilean agriculture.

## 1. Introduction

Pesticides have different physicochemical characteristics, structures, modes of action, and uses in agriculture [1,2]. Depending on their molecular structure, pesticides are classified into different groups, such as organochlorines, organophosphates, neonicotinoids, carbamates, triazine, urines, phenoxyacids, pyrethroids, and triazoles [3]. According to their use in agriculture, pesticides are classified as insecticides, fungicides, nematicides, acaricides, and herbicides [4]. Nowadays, pesticides are widely used in the agricultural industry to reduce the impact of pests, weeds, and diseases in different crops, leading to increases in productivity and a higher quality of crops [5]. However, the improper use of pesticides involves risks for human health, as pesticide residues remain in fresh vegetables [6,7,8], food [9], soil [10], and water bodies [11] after harvest. Hence, the overuse and misuse of pesticides can greatly impact the environment [12,13] and poses a serious risk for human health, since short- or long-term exposure to pesticide residues may cause acute or chronic toxicity [14]. Pesticide residues with different mechanisms of action on humans can be considered as a potential toxicological concern [15]. The consequences of the chronic exposure to organophosphate pesticide residues may include metabolic disorders such as genotoxicity, carcinogenesis [16], neurological disorders [17], and endocrine disruption [18].

Protecting consumers from the exposure to pesticide residues in raw food is a growing concern in Chile [19]. Chemical food safety and security are priorities for the Ministry of Agriculture (MINAGRI) and the Ministry of Health (MINSAL). The Agricultural and Livestock Service (SAG), an entity dependent on the MINAGRI and the Institute of Public Health (ISP), which in turn is dependent on MINSAL, is responsible for ensuring food safety in the country and the compliance of the maximum residue levels (MRLs) approved by the regulation, MRL-762/2011 [20]. This regulation defines the maximum levels of pesticide residues that can be present in food, in accordance with the principles established by the Good Agricultural Practices (GAP) [21]. The use of pesticides with a reasonable certainty of no harm, the pesticide doses administered, and the concentration of pesticide residues which remain in food, as well as laws approved by the government are supervised by SAG [22]. In 2019, a new regulation regarding pesticides containing methamidophos, carbofuran, and azinphos-methyl was legally implemented, enforcing their removal from the Chilean market by June 2021 [23].

The food safety surveillance program in Chile, which is coordinated by the SAG and ISP, was established to regulate the proper use of pesticides in agriculture and their impact on human health. However, aspects such as the potential exposure and risks for human health are not evaluated under the current program. Every year, the national surveillance program evaluates more than 1500 fresh vegetables and fruit samples throughout the country. Since 2017, 15–25% of the samples, mainly fresh vegetables, exceeded the maximum residue levels permitted by the law. Among all the pesticides evaluated in the surveillance, the main transgressions were detected for methamidophos, methomyl, chlorpyrifos, cypermethrin, diazinon, and λ-cyhalothrin. Lettuce, spinach, chard, tomatoes, and peppers were the vegetables with the highest levels of pesticide residues [24,25,26,27,28].

Moreover, the Ministry of Health coordinates the national surveillance of pesticide acute intoxications (REVEP), which informs all notifications in hospitals throughout the country. The REVEP surveillance provided serious evidence of the acute intoxication of farm workers in the main agricultural regions of Chile, including Arica, Coquimbo, Metropolitan, Valparaiso, and Del Libertador Bernardo O’Higgins. The main intoxications were produced by active substances such as methamidophos, diazinon, methomyl, chlorpyrifos, and other pesticides classified as Ia and Ib [29].

The production of tomatoes (*Lycopersicon esculentum*) is susceptible to various diseases and pests [30], which produce qualitative and quantitative damages in the harvest [31]. These vegetables are an important source of carotenoids, minerals, and vitamins which are highly recommended for daily consumption to improve human health and decrease the potential of many human diseases [32]. Tomatoes are one of the main vegetables consumed worldwide. In 2019, the tomato fields in Chile expanded by over 5.328 ha [33]. The tomatoes in Chile are distributed through three main channels: local street markets, supermarkets, and wholesalers. In the Metropolitan Region, more than half of the total population of Chile is concentrated and more than 60% of the total production of vegetables is commercialised and distributed by Lo Valledor S. A., the main wholesaler in Chile. The lack of food safety standards, traceability, good agricultural practices, and enforcement of pesticide residues can be a source of chemical and biological risks to the health of consumers. The aim of this study was to analyse pesticide residues and provide a corresponding dietary risk assessment for tomatoes commercialised in local supermarkets from the Metropolitan Region, Chile.

## 2. Materials and Methods

### 2.1. Sampling 

Fifty-seven samples were collected from local supermarkets of the Metropolitan Region between January and March, 2020. Each sample size consisted of 2 kg of fresh tomatoes wrapped in aluminium foil. The samples were processed using a grinder, and then stored in a flask and frozen at −20 °C, according to the protocol previously described elsewhere [22].

### 2.2. Pesticides

More than 180 active substances used in the agriculture industry that were authorised by the Agricultural and Livestock Services were evaluated in a multiresidue screening program. The pesticides analysed included insecticides, herbicides, fungicides, nematicides, and acaricides, which were the most frequently used pesticides in tomato production throughout the country. The list of pesticides analysed was as follows: abamectin, acephate, acetamiprid, acetochlor, acrinathrin, alachlor, aldicarb, alpha-BHC, alpha-cypermethrin, alpha-HCH, amethrin, atrazine, azinphos-ethyl, azinphos-methyl, azoxystrobin, benalaxyl, beta BHC, bifenthrin, bitertanol, boscalid, bromacil, bromo-phos-ethyl, bromophos-methyl, bromopropylate, buprofezin, butachlor, captan, car-baryl, carbendazim, carbofuran, carbophenotion, cartap, chlorantraniliprole, chlordane, chlorfenapyr, chlorfenvinphos, chlorobenzilate, chlorothalonil, chlorpyrifos-ethyl, chlorpyrifos-methyl, cis-chlordane, cyanazine, cycloate, cyfluthrin, cymoxanil, cyper-methrin, cyproconazol, cyprodinil, cyromazine, DDD-o,p, DDD-p,p’, DDE-p,p’, DDE-o,p’, DDT-o,p, DDT-p,p, delta-BHC, deltamethrin, desmedipham, diazinon, di-chlofluanid, dichlorvos, diclobutrazol, dicloran, dicofol, dieldrin, difenoconazol, disul-foton, dimethoate, dimethomorph, diphenylamine, dithiocarbamates, alfa-endosulfan, beta-endosulfan, endosulfan-sulfate, endrin, EPTC, esfenvalerate, ethion, ethofumesate, fenamiphos, fenarimol, fenbuconazol, fenchlorphos, fenhexamid, fenitrothion, fen-medifam, fenoxycarb, fenpyroximate, fenthion, fenvalerate, fipronil, fluazinam, fludi-oxonil, flufenoxuron, fluquinconazol, flusilazole, fluvalinate, folpet, heptachlor, hepta-chlor epoxide, hexaconazole, hexazinone, imazalil, imidacloprid, indoxacarb, iprodione, lambda-cyhalothrin, lenacil, lindane, linuron, malathion, mefenoxam, metalaxyl, methamidophos, methidathion, methiocarb, methomyl, methoxychlor, metolachlor, metribuzin, mirex, monocrotophos, myclobutanil, napropamid, norflurazon, omethoate, oxamyl, oxyfluorfen, paraquat, parathion-ethyl, parathion-methyl, pebulate, penconazol, pendimethalin, pentachlorobenzene, pentachloronitrobenzene, permethrin, phorate, phosalone, phosmet, pirimicarb, pirimiphos-ethyl, pirimiphos-methyl, prochloraz, procymidone, profenofos, propachlor, propamocarb, propazine, propiconazole, propoxur, pyraclostrobin, pyrazophos, pyridaben, pyrimethanil, quinalphos, quinomethionate, quinoxyfen, rotenone, simazine, spirodiclofen, tebuconazole, tebufenozide, ter-buthylazine, tetraconazole, tetradifon, thiabendazole, thiacloprid, thiamethoxam, thio-cyclam, thiodicarb, thiophanate-methyl, thiuram, tolylfluanid, triadimefon, triadimenol, triazophos, trichlorfon, trifloxystrobin, triflumizole, trifluralin and vinclozolin. Analytical grade pesticides standards (over 99% purity) were obtained from Sigma-Aldrich (Saint Louis, MO, USA), HPLC (Cunnersdorf, Deutschland), and Chem Service (West Chester, PA, USA). Solvents and chemicals were obtained from Merck (Darmstadt, Germany) [22]. QuEChERS extraction systems were purchased from UCT (Bristol, PA, USA).

### 2.3. Pesticide Analysis and Quality Assurance

Pesticide extraction was performed using the QuEChERS method [34], as previously described elsewhere [21,22]. Briefly, 10 g of tomato sample were extracted with acetonitrile and extraction mix (4 g MgSO_4_; 1 g NaCl and 1.5 g citrate), manually shaken and centrifugated. The supernatant was transferred and the clean-up step was performed using 900 mg MgSO_4_, 150 mg PSA and 150 mg C18, manually shaken and centrifugated, and the extract was transferred to a vial until analysis at −20 °C. Pesticides residues were analyzed by GC or HPLC according to their functional groups, volatility and derivatisation properties. The concentration of organophosphates was quantified using a GC-NPD Agilent 7890 with autosampler (Santa Clara, CA, USA). The concentration of halogenated proteins was quantified using a GC-electron capture detector (Thermo Scientific Trace-Ultra) with an autosampler (Waltham, MA, USA) and Perkin Elmer Auto-System XL (Waltham, MA, USA). The concentration of dithiocarbamates was determined using distillation and quantification with a Thermo 10VIS spectrophotometer (Thermo Scientific Inc., Madison, WI, USA). Results were expressed as mg of carbon disulphide (CS_2_) per kg. The concentration of methyl-carbamates was quantified using HPLC with a Merck Hitachi LaChrom D-7000-autosampler (Dartford, United Kingdom) coupled to a fluorescence detector and a reaction pump (655A-B) from Merck Hitachi (Dartford, United Kingdom). To determine the concentration of imidacloprid and carbendazim, a HPLC system Merck Hitachi D-6000 with a UV detector was used (Burladingen, Germany) [21,22]. The concentration of dithiocarbamates was determined using distillation and quantification with a Thermo 10VIS spectrophotometer (Thermo Scientific Inc., Madison, WI, USA). Results were expressed as mg of carbon disulphide (CS2) per kg [21,22]. 

Quality assurance was carried out following the SANTE 12682/2019 guidelines. Standard ISO/IEC 17025:2017 of the Accredited Laboratory of Pesticide Residues was implemented. The λ-cyhalothrin, myclobutanil, buprofezin, indoxacarb, pyrimethanil, difenoconazole, azoxystrobin, boscalid, chlorfenapyr and chlorpyrifos were analysed by gas chromatography ECD detector. Additionally, methamidophos and acetamiprid were analysed by GC-NPD detector. The imidacloprid was analysed by liquid chromatography HPLC-DAD and methomyl by HPLC-FL detector. The accuracy was expressed as percentage of recovery, and the precision as repeatability both were used for the validation process [21,22]. Tomato blank samples (free of pesticides) were used to perform the quality assurance. The recovery was studied at a concentration of 20 µg/kg and the precision was determined as the relative standard deviation (RSD). The Limit of detection (LOD) was determined as the signal-to-noise ratio and Limit of Quantification (LOQ) as the lowest concentration quantifiable with acceptable recoveries. Calibration curves were evaluated in blank tomato samples between 10 and 320 µg/kg for the detected pesticides residues.

### 2.4. Compliance of Chilean Maximum Residue Levels

The results were verified under the current regulation of MRLs of Chilean RES 762/2011, which was mandatory for all fresh food commercialised in supermarkets under the national regulation RSA Nº 977/1996. In addition, we checked that the pesticide residues detected in the tomato samples were authorised by the Agricultural and Livestock Service.

### 2.5. Dietary Risk Assessment

The levels of active substances were included in the dietary risk assessments. Two genders were analysed according to the data obtained at national and international levels. Body weight and age groups were obtained from a national health survey [21,22,23,24,25,26,27,28,29,30,31,32,33,34,35]. The Chilean tomato consumption and the international daily tomato consumption were set to 120 and 10.5 g/day, respectively [22].

The Acceptable Daily Intakes (ADI) for the pesticides evaluated in this study were: 0.025 mg/kg bw/d for acetamiprid [36]; 0.010 mg/kg bw/d for buprofezin [37]; 0.030 mg/kg bw/d for chlorfenapyr [38]; 0.001 mg/kg bw/d for chlorpyrifos [39]; 0.060 mg/kg bw/d for imidacloprid [40]; 0.060 mg/kg bw/d for λ-cyhalothrin [41]; 0.001 mg/kg bw/d for methamidophos [42]; 0.1 mg/kg bw/d for azoxystrobin [43]; 0.010 mg/kg bw/d for difenoconazole [44]; 0.025 mg/kg bw/d for myclobutanil [45]; 0.005 mg/kg bw/d for indoxacarb [46]; 0.040 mg/kg bw/d for boscalid [47]; 0.003 mg/kg bw/d for methomyl [48]; and 0.170 mg/kg bw/d for pyrimethanil [49]. 

The estimated daily intake (EDI) was evaluated by multiplying the levels of active substances by food consumption divided by body weight. The hazard quotients were calculated as HQ = EDI/ADI × 100 [50]. The chronic hazard index (cHI) [51] was calculated for all the pesticides with similar health effects, as described in the Pesticide Properties Database (PPDB) website of the University of Hertfordshire. The pesticides were classified as acetyl cholinesterase inhibitors (chlorpyrifos, methamidophos, methomyl); neurotoxicant (chlorpyrifos, methamidophos, methomyl), respiratory tract irritants (λ-cyhalothrin, methomyl); skin irritants (acetamiprid, azoxystrobin, difenoconazole); skin sensitisers (λ-cyhalothrin, indoxacarb); and eye irritants (λ-cyhalothrin, azoxystrobin, difenoconazole, myclobutanil, methomyl). A cHI > 100 indicated that the exposure induced obvious toxic effects, whereas a cHI < 100 indicated that consumption was considered acceptable [50].

## 3. Results

The samples were analysed to quantify multi-pesticide residues and evaluate their compliance with MRL established by the Chilean regulations and their associated dietary risk assessments. All the pesticides reported in this study were previously validated following the guidelines of SANTE 12682/2019 and ISO/IEC 17025:2017 of the accredited laboratory of pesticide residues at National Institute of Agriculture. The recoveries were described as follows: λ-cyhalothrin 99.9%; buprofezin 95.2%; indoxacarb 101.9%; chlorfenapyr 101%; chlorpyrifos 95.5%; methamidophos 92.2%; acet-amiprid 107.3%; imidacloprid 100.5%; methomyl 103.6%; pyrimethanil 89.7%; difenoconazole 99.4%; azoxystrobin 99.5%; boscalid 101.2%; and myclobutanil 92.4%. On the other hand, the RSD % values ranged between 2.3–10.2%.

### 3.1. Screening of Pesticide Residues and Their Compliance of MRL

In our study, pesticide residues were analysed in 57 samples of tomatoes to assess their compliance with national regulations. The first screening detected the presence of different residues of λ-cyhalothrin, buprofezin, indoxacarb, chlorfenapyr, chlorpyrifos, methamidophos, acetamiprid, imidacloprid, methomyl, pyrimethanil, difenoconazole, azoxystrobin, boscalid, and myclobutanil. Of the total samples evaluated, 39% of samples were free of pesticide residues, 35% contained one residue, 17% contained two residues, and 39% contained three or more residues. The residue concentration in 9% of the contaminated samples was above the MRL. Interestingly, all samples containing methamidophos had concentrations above the corresponding MRL.

### 3.2. Multi Pesticide Residues Analysis

All the pesticides detected in this study were authorised by the Chilean authorities for their use in tomato production. More than 60% of pesticide residues detected in the samples were insecticides: λ-cyhalothrin, buprofezin, indoxacarb, chlorfenapyr, chlorpyrifos, methamidophos, acetamiprid, imidacloprid, and methomyl (Table 1). The main insecticide detected was acetamiprid, which was present in 21% of the total samples. In addition, the main fungicide detected was difenoconazole, present in 11.5% of the samples. Pyrimethanil had the highest mean concentration: 0.23 mg/kg. The mean concentration of methamidophos was 0.12 mg/kg, which was 12 times higher than the Chilean MRL. Ac-cording to these results, methamidophos represented the highest transgression in this study. This result was in accordance with previous studies.

### 3.3. Dietary Risk Assessment 

The dietary risk assessment was conducted on two different age groups, including males and females, using national and international data. The EDI was calculated as described above. The consumption level was set up to 10.5 g/day, and 120 g/day for the WHO and Chile, respectively. In all the scenarios evaluated, the risk assessment determined for Chile was higher than that of the WHO (Table 2). In general, the EDI obtained with the Chile model was 11 times higher than that of the WHO model. In addition, the EDI determined for females (WOMAN model) was higher than that determined for males (MEN model) (Table 2a and Table 2b, respectively). In the male group, the highest EDI values corresponded to the active substances: pyrimethanil, chlorfenapyr, methamidophos, ac-etamiprid, and myclobutanil (Table 2a). In the Chile model (Table 2b), the highest values of pyrimethanil were detected as 4.5 × 10^−4^, 4.0 × 10^−4^, 3.9 × 10^−4^ and 4.2 × 10^−4^ mg/kg bw/day. The highest EDI for chlorfenapyr in the age group 15–24 years was 3.9 × 10^−4^ mg/kg bw/day, and for methamidophos: 2.4 × 10^−4^ mg/kg bw/d. For the Chile model (Table 2d), in all age groups, the EDI of pyrimethanil was the highest, with values of 4.5 × 10^−4^, 4.1 × 10^−4^, 4.0 × 10^−4^ and 4.2 × 10^−4^ mg/kg bw/d, respectively. The highest EDI for chlorfenapyr in the age group 15–24 was 3.9 × 10^−4^ mg/kg bw/day. Moreover, the highest EDI for methamidophos was 2.4 × 10^−4^ mg/kg bw/day. In the Chile model, we calculated the cHI for all pesticide residues with similar health effects according to the information described in the Pesticide Properties Database (PPDB) from the University of Hertfordshire (Table 3). The values of cHI were highest for the organophosphate active substance: chlorpyrifos, methamidophos, and methomyl. The values obtained for methamidophos and chlorpyrifos could induce chronic toxic effects d. In this study, the highest cHI for both the acetyl cholinesterase inhibitors and the neurotoxicant effects were 27.9 and 28, respectively.

## 4. Discussion

The limits of detection in our work ranged from 5–10 µg/kg and the limit of quantification was between 10–20 µg/kg. Supporting our findings, in a validation study the LOD ranged from 2.35 µg/kg for benthiavalicarb to 6.49 µg/kg for allethrin in fresh tomatoes [51]. On another hand, the recovery rates of our study ranged from 89.7–107.3% for all the pesticides quantified according to the range proposed by the SANTE guide from 0.1 to 19.6%. Supporting our results, similar results reported a range from 80.1 and 112% for 24 pesticide residues in tomatoes marketed in Colombia [52]. In addition, results from 72–116% were obtained in multiclass pesticide residues in tomato samples collected from different markets of Iran [53]. The RSD values were as follows: λ-cyhalothrin 2.7%; buprofezin 3.6%; indoxacarb 3.3%; chlorfenapyr 2.9%; chlorpyrifos 4.3%; methamidophos 4.5%; acetamiprid 2.3%; imidacloprid 10.2%; methomyl 9.5%; pyrimethanil 6.1%; difenoconazole 4.5%; azoxystrobin 3.9%; boscalid 3.1%; and myclobutanil 5.8%. These results showed that the results and their accuracy were positive and the laboratory competences were confirmed. Comparing the results obtained in this work to other studies, the RSD values from 2.1–17.9% were obtained for pesticide residues in tomatoes in Turkey [54].

Previous surveillance studies reported the existence of pesticide residues in local markets worldwide. A recent study analysed tomato samples (*n* = 20) from the local markets of Majmaah Province, Saudi Arabia, and determined that 27% of the analysed samples contained pesticide residues, cypermethrin in most of the cases [55]. Moreover, a surveillance study of organophosphates in Northern Thailand was performed using 160 samples of vegetables, including tomatoes from local markets. They described a rate of chlorpyrifos residues of 33.8% in all the samples detected [56]. Finally, a monitoring study in tomatoes marketed in Bogota, Colombia (*n* = 400) discovered at least one pesticide in 70.5% of the total samples evaluated. In this study, the most frequently detected active substances were pyrimethanil, carbendazim, dimethomorph, and acephate [52].

Based on our results and the previously reported literature, we considered that methamidophos residues posed a potential risk to human health [21,22]. A previous study conducted on the residues in tomatoes from Iranian markets (*n* = 150) reported the presence of both chlorpyrifos and diazinon residues above the MRL [53]. Similar results were obtained in tomatoes from Ghana (*n* = 20), in which the residues of methamidophos, malathion, and dimethoate exceeded the corresponding MRL [57]. A surveillance study in vegetables in Saudi Arabia, including tomatoes (*n* = 26), reported a high frequency of methomyl, imidacloprid, metalaxyl, and cyproconazole residues [58]. In Argentina, pesticide residues were evaluated in several vegetables, including tomatoes from domestic markets (*n* = 10), and found that 65% of the total samples were contaminated and that 20% were above the MRL [59]. A surveillance study in Kuwait also analysing tomatoes (*n* = 16), determined that 21% of samples had a pesticide residue concentration above the MRL. The pesticides more frequently detected in this study were imidacloprid, deltamethrin, cypermethrin, malathion, acetamiprid, monocrotophos, chlorpyrifos, and diazinon, all of which exceeded their corresponding MRLs [60]. In a study including tomato samples (*n* = 17) from the Burkina Faso market, 36% of the pesticide-containing samples exceeded the MRL. The main residues detected were acetamiprid, carbofuran, chlorpyrifos, λ-cyhalothrin, dieldrin, imidacloprid, and profenofos. According to a health risk assessment included in this study, chlorpyrifos and λ-cyhalothrin posed a threat to human health [61]. A study conducted in Canada on 133 samples of vegetables, including 17 samples of tomatoes, reported that 47% of samples were above the limit of detection for at least one pesticide. Among all the pesticide residues detected, the active substances imidacloprid, acetamiprid, and clothianidin were the most recurrent [62]. Furthermore, similar results were reported in tomatoes obtained from the Jordan Valley, with transgressions of chlorothalonil and daminozide [63]. Finally, another surveillance study determined that 61% of the tomatoes cultivated in greenhouses in the Mediterranean region of Turkey contained the active substances: chlorpyrifos methyl, cyfluthrin, deltamethrin, and acetamiprid [54].

In a study from Saudi Arabia, acetamiprid was the most commonly detected pesticide residue in tomatoes from supermarkets (*n* = 10) [64]. In a study conducted on Nepalese tomatoes (*n* = 32), all the samples showed pesticide residues, mainly chlorpyrifos and carbendazim [65]. Additionally, parathion, malathion, endosulfan, dieldrin, and DDT concentrations above the MRL were detected in a study of Tanzanian tomato samples (*n* = 17) [66]. In a study conducted on tomato samples from Valencia (*n* = 90), 13.3% of the samples had carbendazim, 12.2% had chlorpyrifos, 6.7% had cypermethrin, and 4.4% had λ-cyhalothrin [67]. A surveillance study in Senegal on vegetables including tomatoes (*n* = 57) reported that 65% of the samples had active substances such as DDT, dimethoate, and λ-cyhalothrin [68]. Finally, a study conducted on Turkish vegetables, including tomatoes (*n* = 177), detected pesticide residues in 67% of the tomato samples evaluated, of which 14% had pesticide residues concentrations above the MRL. The main pesticides detected in this study were acetamiprid (9 samples > MRL), carbendazim (1 sample > MRL), oxamyl (2 sample > MRL), tebuconazole (6 samples > MRL), azoxystrobin, boscalid, pyridaben, and fludioxonil [69].

Several previous studies reported that the EDI of pesticide residues in tomatoes were higher than our results. A study conducted on tomatoes from Northeast China (*n* = 36) reported that the EDIs of methamidophos, dichlorvos, diazinon and omethoate were 4.2-fold, 1.7-fold, 1.2-fold, and 4.1-fold higher than the respective AIDs for adults. The maximum EDIS for children reported in this study for methamidophos, dichlorvos, diazinon and omethoate were 3.2-fold, 1.3-fold, 0.96-fold and 3.17-fold higher than their corresponding ADI [70]. A study conducted on tomatoes from Zhejiang, China (*n* = 237), reported that the EDI of chlorpyrifos and cypermethrin in a group of children (2 to 6 years old) was 48.9% and 31.8% of ADI, respectively [71]. In contrast with these results, another study assessing the presence of pesticide residues in several vegetables in Zambia, including tomatoes (*n* = 9), reported an EDI below the ADI estimated by the World Health Organization and the Food and Agriculture Organization (FAO) [72]. Another study assessed the pesticide concen-trations in tomato samples from Tanzania (*n* = 50), and reported that the EDIs for chlorpyrifos, permethrin, and ridomil were higher than the values permitted, indicating that consumption of fresh tomatoes could pose health risks to the consumer [73]. Furthermore, a study of pesticide residues in tomatoes from Kazakhstan (*n* = 44) reported EDI values ranging from 0.01% of the ADI established for pyrimethanil, to 12.05% of the ADI established for λ-cyhalothrin. The most critical pesticides were triazophos and flusilazole, contributing 70.8% and 42.5% to the cHI [74].

**Figure 1:**
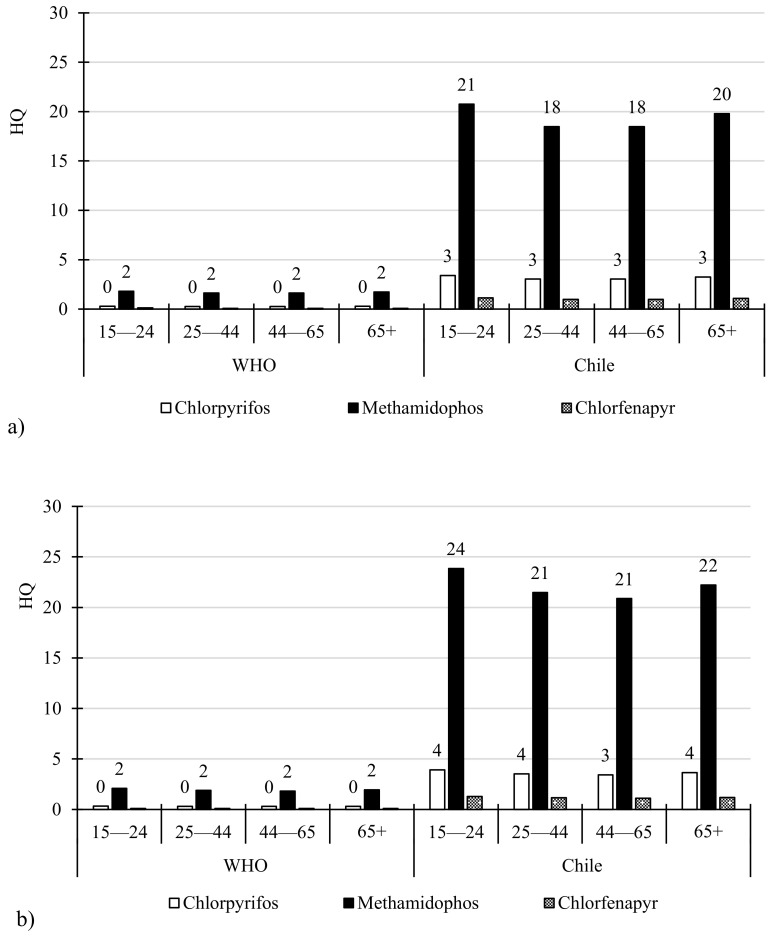
Hazard Quotients (HQ) for all the scenarios in fresh tomatoes from local supermarkets of the Metropolitan Region, Chile: (**a**) Men; (**b**) Woman.

The HQs for all scenarios are described in Figure 1. Methamidophos had the highest value. Methamidophos showed the highest value in the Chilean model, age group 15–24 (23.8 in the WOMAN model and 20.8 in the MEN model). As shown in Figure 1a, the HQ for MEN decreased in the following order: methamidophos > chlorpyrifos > buprofezin > difenoconazole > myclobutanil > λ-cyhalothrin. Moreover, as shown in Figure 1b, the HQs for the WOMAN model decreased in the same order as that described in the model, MEN. A study conducted on Nepalese tomatoes assessed the HQ and cHI in adolescents and adults, and reported similar finding to our study [65].

A previous study carried out on 19 tomato samples reported a high exposure to chlorpyrifos and ethion, supporting our findings. In this study, the HI was approximately 100% of the ADI, and chlorpyrifos was reported as a risk for adults [54]. HQ > 1 was observed for the active substances profenofos, triazophos, dimethoate, omethoate, chlorpyrifos, and carbendazim with high HQs [68]. Contrasting results were reported in tomatoes cultivated in greenhouses from the Turkish Mediterranean region, with 61% of samples containing chlorpyrifos methyl, cyfluthrin, deltamethrin, or acetamiprid. All the pesticides mentioned showed an HI of 9.5% for adults and 11.02 for children (3 to >10 years), mainly owing to the presence of chlorpyrifos [66]. Moreover, a study, assessing the health risk of tomatoes from Kazakhstan, reported triazophos and flusilazole residues, indicating that pesticide residues could be considered a public health issue [74]. In addition, some samples of tomato with an HI higher than 1 for chlorothalonil were reported and could pose a threat to children’s health [75].

## 5. Conclusions

Chilean supermarkets conduct internal testing programmes on pesticide residues. The aim of this private surveillance is to identify non-compliances with the Maximum Residue Levels set by the Chilean government. If the pesticide residues levels in fresh tomatoes are above the MRL, the supermarkets force the farmers to reduce the number of pesticide applications and to improve the use of good agricultural practices. Therefore, supermarkets should implement a larger sampling test to cover a higher number of tomato samples and identify hazardous pesticides such as methamidophos, chlorpyriphos and methomyl. In addition, the Chilean authorities should increase the effort for testing fresher tomato samples for national consumption in supermarkets of the Metropolitan Region. 

In our study, 9% of the total samples evaluated showed concentrations of pesticide residues above the Maximum Residue Levels of pesticides permitted in Chile. Based on the results obtained, methamidophos, chlorpyriphos and methomyl, which are internationally considered to be highly hazardous pesticides by the Food and Agriculture Organization and the World Health Organization of the United Nations, should be restricted in their use on tomatoes marketed in Chile. However, the main limitations of our study are the lack of consumption frequency and body weight data for children. Further dietary consumption studies are necessary for conducting a health risk assessment in Chile.

## Figures and Tables

**Table 1:** Distribution and pesticide residues levels detected in fresh tomatoes.

Pesticide	Category	Frecuency	% from the Total Pesticide Detected	Mean (mg/kg)	Range min–max, (mg/kg)	Maximun Residue Level MRL (mg/kg)
λ-cyhalothrin	I	5	8.2	0.01	0.0025–0.02	0.10
Buprofezin	I	5	8.2	0.05	0.027–0.078	1.00
Indoxacarb	I	5	8.2	0.01	0.0025–0.022	0.50
Chlorfenapyr	I	6	9.8	0.20	0.043–0.589	1.00
Chlorpyrifos	I	3	4.9	0.02	0.022–0.036	0.50
Methamidophos	I	5	8.2	0.12	0.026–0.459	0.01
Acetamiprid	I	13	21.3	0.09	0.015–0.49	0.20
Imidacloprid	I	2	3.3	0.05	0.0025–0.045	0.50
Methomyl	I	1	1.6	0.05	0.0025	0.50
Pyrimethanil	F	1	1.6	0.23	0.0025–0.233	0.70
Difenoconazole	F	7	11.5	0.05	0.013–0.151	0.50
Azoxystrobin	F	1	1.6	0.00	0.0025	3.00
Boscalid	F	6	9.8	0.03	0.022–0.091	3.00
Myclobutanil	F	1	1.6	0.09	0.0025–0.085	0.30

I—Insecticide, F—Fungicide.

**Table 2:** Estimated Daily Intake (mg/kg bw/d) for different gender, age groups, national and international scenarios: a) Men; b) Woman.

**(a)** **Pesticide**	**Acceptable Daily Intake** **(mg/kg)**	**(a) World Health** **Organization**				**(b) Chile**			
		**15–24**	**25–44**	**44–65**	**65+**	**15–24**	**25–44**	**44–65**	**65+**
Acetamiprid	2.5 × 10^−2^	1.3 × 10^−5^	1.2 × 10^−5^	1.2 × 10^−5^	1.3 × 10^−5^	1.5 × 10^−4^	1.4 × 10^−4^	1.4 × 10^−4^	1.4 × 10^−4^
Buprofezin	1.0 × 10^−2^	7.3 × 10^−6^	6.5 × 10^−6^	6.5 × 10^−6^	7.0 × 10^−6^	8.3 × 10^−-5^	7.4 × 10^−5^	7.4 × 10^−5^	8.0 × 10^−5^
Chlorfenapyr	3.0 × 10^−2^	3.0 × 10^−5^	2.6 × 10^−5^	2.6 × 10^−5^	2.8 × 10^−5^	3.4 × 10^−4^	3.0 × 10^−4^	3.0 × 10^−-4^	3.2 × 10^−4^
Chlorpyrifos	1.0 × 10^−3^	3.0 × 10^−6^	2.7 × 10^−6^	2.7 × 10^−6^	2.8 × 10^−6^	3.4 × 10^−5^	3.0 × 10^−5^	3.0 × 10^−5^	3.3 × 10^−5^
Imidacloprid	6.0 × 10^−2^	6.7 × 10^−6^	5.9 × 10^−6^	5.9 × 10^−6^	6.4 × 10^−6^	7.6 × 10^−5^	6.8 × 10^−5^	6.8 × 10^−5^	7.3 × 10^−5^
λ-cyhalothrin	2.5 × 10^−3^	1.5 × 10^−6^	1.3 × 10^−6^	1.3 × 10^−6^	1.4 × 10^−6^	1.7 × 10^−5^	1.5 × 10^−5^	1.5 × 10^−5^	1.6 × 10^−5^
Methamidophos	1.0 × 10^−3^	1.8 × 10^−5^	1.6 × 10^−5^	1.6 × 10^−5^	1.7 × 10^−5^	2.1 × 10^−4^	1.8 × 10^-4^	1.8 × 10^−4^	2.0 × 10^−4^
Azoxystrobin	1.0 × 10^−1^	3.7 × 10^−7^	3.3 × 10^−7^	3.3 × 10^−7^	3.5 × 10^−7^	4.2 × 10^−6^	3.8 × 10^−6^	3.8 × 10^−4^	4.0 × 10^−6^
Difenoconazole	1.0 × 10^−2^	6.8 × 10^−6^	6.1 × 10^−6^	6.1 × 10^−6^	6.5 × 10^−6^	7.8 × 10^−5^	7.0 × 10^−5^	7.0 x 10^-5^	7.5 × 10^−5^
Miclobutanil	2.5 × 10^−2^	1.3 × 10^−5^	1.1 × 10^−5^	1.1 × 10^−5^	1.2 × 10^−5^	1.4 × 10^−4^	1.3 × 10^−4^	1.3 × 10^−4^	1.4 × 10^−4^
Indoxacarb	5.0 × 10^−3^	9.5 × 10^−7^	8.4 × 10^−7^	8.4 × 10^−7^	9.0 × 10^−7^	1.1 × 10^−5^	9.6 × 10^−6^	9.6 × 10^−6^	1.0 × 10^−5^
Boscalid	4.0 × 10^−-2^	4.8 × 10^−6^	4.3 × 10^−6^	4.3 × 10^−6^	4.6 × 10^−6^	5.5 × 10^−5^	4.9 × 10^−5^	4.9 × 10^−5^	5.3 × 10^−5^
Methomyl	2.5 × 10^−3^	3.7 × 10^−7^	3.3 × 10^−7^	3.3 × 10^−7^	3.5 × 10^−7^	4.2 × 10^−6^	3.8 × 10^−6^	3.8 × 10^−6^	4.0 × 10^−6^
Pirimetanil	1.7 × 10^−1^	3.4 × 10^−5^	3.1 × 10^−5^	3.1 × 10^−5^	3.3 × 10^−5^	3.9 × 10^−4^	3.5 × 10^−4^	3.5 × 10^−4^	3.8 × 10^−4^
**(b)** **Pesticide**	**Acceptable Daily Intake** **(mg/kg)**	**(c)** **World Health** **Organization**				**(d) Chile**			
		**15–24**	**25–44**	**44–65**	**65+**	**15–24**	**25–44**	**44–65**	**65+**
Acetamiprid	2.5 × 10^−2^	1.5 × 10^−5^	1.4 × 10^−5^	1.3 × 10^−5^	1.4 × 10^−5^	1.7 × 10^−4^	1.6 × 10^−4^	1.5 × 10^−4^	1.6 × 10^−4^
Buprofezin	1.0 × 10^−2^	8.4 × 10^−6^	7.6 × 10^−6^	7.3 × 10^−6^	7.8 × 10^−6^	9.6 × 10^−5^	8.6 × 10^−5^	8.4 × 10^−5^	8.9 × 10^−5^
Chlorfenapyr	3.0 × 10^−2^	3.4 × 10^−5^	3.1 × 10^−5^	3.0 × 10^−5^	3.2 × 10^−5^	3.9 × 10^−4^	3.5 × 10^−4^	3.4 × 10^−4^	3.6 × 10^−4^
Chlorpyrifos	1.0 × 10^−3^	3.4 × 10^−6^	3.1 × 10^−6^	3.0 × 10^−6^	3.2 × 10^−6^	3.9 × 10^−5^	3.5 × 10^−5^	3.4 × 10^−5^	3.7 × 10^−5^
Imidacloprid	6.0 × 10^−2^	7.6 × 10^−6^	6.9 × 10^−6^	6.7 × 10^−6^	7.1 × 10^−6^	8.7 × 10^−5^	7.9 × 10^−5^	7.6 × 10^−5^	8.1 × 10^−5^
λ-cyhalothrin	2.5 × 10^−3^	1.7 × 10^−6^	1.5 × 10^−6^	1.5 × 10^−6^	1.6 × 10^−6^	1.9 × 10^−5^	1.7 × 10^−5^	1.7 × 10^−5^	1.8 × 10^−5^
Methamidophos	1.0 × 10^−3^	2.1 × 10^−5^	1.9 × 10^−5^	1.8 × 10^−5^	1.9 × 10^−5^	2.4 × 10^−4^	2.1 × 10^−4^	2.1 × 10^−4^	2.2 × 10^−4^
Azoxystrobin	1.0 × 10^−1^	4.2 × 10^−7^	3.8 × 10^−6^	3.7 × 10^−7^	4.0 × 10^−7^	4.9 × 10^−6^	4.4 × 10^−6^	4.2 × 10^−6^	4.5 × 10^−6^
Difenoconazole	1.0 × 10^−2^	7.9 × 10^−6^	7.1 × 10^−5^	6.9 × 10^−6^	7.3 × 10^−6^	9.0 × 10^−5^	8.1 × 10^−5^	7.9 × 10^−5^	8.4 × 10^−5^
Miclobutanil	2.5 × 10^−2^	1.4 × 10^−5^	1.3 × 10^−5^	1.3 × 10^−5^	1.3 × 10^−5^	1.7 × 10^−4^	1.5 × 10^−4^	1.4 × 10^−-4^	1.5 × 10^−4^
Indoxacarb	5.0 × 10^−3^	1.1 × 10^−6^	9.8 × 10^−7^	9.5 × 10^−7^	1.0 × 10^−6^	1.2 × 10^−5^	1.1 × 10^−5^	1.1 × 10^−5^	1.2 × 10^−5^
Boscalid	4.0 × 10^−2^	5.6 × 10^−6^	5.0 × 10^−6^	5.2 × 10^−6^	5.2 × 10^−6^	6.4 × 10^−5^	5.7 × 10^−5^	5.9 × 10^−5^	5.9 × 10^−5^
Methomyl	2.5 × 10^−3^	4.2 × 10^−7^	3.8 × 10^−7^	3.7 × 10^−7^	4.0 × 10^−7^	4.9 × 10^−6^	4.4 × 10^−6^	4.2 × 10^−6^	4.5 × 10^−6^
Pirimetanil	1.7 × 10^−1^	4.0 × 10^−5^	3.6 × 10^−5^	3.5 × 10^−5^	3.7 × 10^−5^	4.5 × 10^−4^	4.1 × 10^−4^	4.0 × 10^−4^	4.2 × 10^−4^

**Table 3:** Chronic Hazard Index for the for all the scenarios in fresh tomatoes from local supermarkets of Metropolitan region, Chile: (a) Men-WHO; (b) Men-Chile; (c) Woman-WHO; (d) Woman-Chile.

Scenario	Age Group	Reproduction Development Effects	Acetyl Cholinesterase Inhibitor	Neurotoxicant	Respiratory Tract Irritant	Skin Irritant	Skin Sensitiser	Eye Irritant
(a) Men WHO	15–24	0.01	2.13	2.13	0.07	0.12	0.08	0.19
	25–44	0.01	1.90	1.90	0.07	0.11	0.07	0.17
	44–65	0.01	1.90	1.90	0.07	0.11	0.07	0.17
	65+	0.01	2.03	2.04	0.07	0.12	0.07	0.18
(b) Men Chile	15–24	0.13	24.33	24.38	0.84	1.39	0.89	2.20
	25–44	0.11	21.68	21.72	0.75	1.24	0.79	1.96
	44–65	0.11	21.68	21.72	0.75	1.24	0.79	1.96
	65+	0.12	23.22	23.27	0.80	1.33	0.85	2.10
(c) Woman WHO	15–24	0.01	2.45	2.45	0.08	0.14	0.09	0.22
	25–44	0.01	2.20	2.21	0.08	0.13	0.08	0.20
	44–65	0.01	2.14	2.15	0.07	0.12	0.08	0.19
	65+	0.01	2.28	2.28	0.08	0.13	0.08	0.21
(d) Woman Chile	15–24	0.15	27.95	28.01	0.96	1.60	1.02	2.53
	25–44	0.13	25.18	25.23	0.87	1.44	0.92	2.28
	44–65	0.13	24.47	24.52	0.84	1.40	0.89	2.21
	65+	0.14	26.06	26.11	0.90	1.49	0.95	2.35

## Data Availability

Not applicable.
